# Diagnostic Accuracy of CT for Metastatic Epidural Spinal Cord Compression

**DOI:** 10.3390/cancers14174231

**Published:** 2022-08-31

**Authors:** James Thomas Patrick Decourcy Hallinan, Shuliang Ge, Lei Zhu, Wenqiao Zhang, Yi Ting Lim, Yee Liang Thian, Pooja Jagmohan, Tricia Kuah, Desmond Shi Wei Lim, Xi Zhen Low, Ee Chin Teo, Nesaretnam Barr Kumarakulasinghe, Qai Ven Yap, Yiong Huak Chan, Jiong Hao Tan, Naresh Kumar, Balamurugan A. Vellayappan, Beng Chin Ooi, Swee Tian Quek, Andrew Makmur

**Affiliations:** 1 Department of Diagnostic Imaging, National University Hospital, 5 Lower Kent Ridge Rd, Singapore 119074, Singapore; 2 Department of Diagnostic Radiology, Yong Loo Lin School of Medicine, National University of Singapore, 10 Medical Drive, Singapore 117597, Singapore; 3 Department of Computer Science, School of Computing, National University of Singapore, 13 Computing Drive, Singapore 117417, Singapore; 4 National University Cancer Institute, NUH Medical Centre (NUHMC), Levels 8–10, 5 Lower Kent Ridge Road, Singapore 119074, Singapore; 5 Biostatistics Unit, Yong Loo Lin School of Medicine, 10 Medical Drive, Singapore 117597, Singapore; 6 University Spine Centre, Department of Orthopaedic Surgery, National University Health System, 1E, Lower Kent Ridge Road, Singapore 119228, Singapore; 7 Department of Radiation Oncology, National University Cancer Institute Singapore, National University Hospital, Singapore 119074, Singapore

**Keywords:** metastatic spinal cord compression, epidural spinal cord compression, metastatic epidural spinal cord compression, spinal metastatic disease, spinal metastases classification, magnetic resonance imaging, MRI, computed tomography, CT, spine oncology study group, Bilsky classification

## Abstract

**Simple Summary:**

Early diagnosis of metastatic epidural spinal cord compression (MESCC) is vital to prevent paralysis. Staging CT scans are performed routinely in cancer patients and could detect MESCC earlier. In this study, we assessed the performance of the original radiologist report for MESCC grading compared to three radiologists performing detailed MESCC evaluation using dedicated CT windows. Two expert radiologists provided the reference standard using MRI scans performed within 30 days. For normal/none versus low/high-grade MESCC per CT scan, all radiologists demonstrated almost perfect agreement with kappa values ranging from 0.866 (95% CI 0.787–0.945) to 0.947 (95% CI 0.899–0.995), compared to only slight agreement for the reports (kappa = 0.095, 95% CI−0.098–0.287). Radiologists also showed high sensitivities ranging from 91.51 (95% CI 84.49–96.04) to 98.11 (95% CI 93.35–99.77), compared to 44.34 (95% CI 34.69–54.31) for the reports. In conclusion, a dedicated radiologist review for MESCC on CT showed improved performance compared to the original report (current standard of care).

**Abstract:**

Background: Early diagnosis of metastatic epidural spinal cord compression (MESCC) is vital to expedite therapy and prevent paralysis. Staging CT is performed routinely in cancer patients and presents an opportunity for earlier diagnosis. Methods: This retrospective study included 123 CT scans from 101 patients who underwent spine MRI within 30 days, excluding 549 CT scans from 216 patients due to CT performed post-MRI, non-contrast CT, or a gap greater than 30 days between modalities. Reference standard MESCC gradings on CT were provided in consensus via two spine radiologists (11 and 7 years of experience) analyzing the MRI scans. CT scans were labeled using the original reports and by three radiologists (3, 13, and 14 years of experience) using dedicated CT windowing. Results: For normal/none versus low/high-grade MESCC per CT scan, all radiologists demonstrated almost perfect agreement with kappa values ranging from 0.866 (95% CI 0.787–0.945) to 0.947 (95% CI 0.899–0.995), compared to slight agreement for the reports (kappa = 0.095, 95%CI −0.098–0.287). Radiologists also showed high sensitivities ranging from 91.51 (95% CI 84.49–96.04) to 98.11 (95% CI 93.35–99.77), compared to 44.34 (95% CI 34.69–54.31) for the reports. Conclusion: Dedicated radiologist review for MESCC on CT showed high interobserver agreement and sensitivity compared to the current standard of care.

## 1. Introduction

Vertebral metastases are seen in up to 40% of patients with cancer, and approximately 20% of such patients progress to metastatic epidural spinal cord compression (MESCC) [1]. Early diagnosis of MESCC is vital to initiate appropriate therapy and prevent permanent neurological dysfunction [2,3]. In addition, with the continuing development of more effective and targeted systemic therapies, the incidence of vertebral metastases and their complications are expected to increase [4,5,6,7].

The spinal column is the most common site for bone metastases, and the most frequent sites of MESCC are along the thoracic vertebrae (70%), lumbar vertebrae (20%), and cervical vertebrae (10%) [8]. Clinical diagnosis of MESCC can be challenging as patients may be asymptomatic or present with non-specific symptoms, which can overlap with those already present from degenerative spine disease. Symptoms preceding paresis or paralysis in MESCC include pain (change in frequency or characteristics), reduced mobility, and altered sensations (e.g., temperature sensations or tingling). Given these clinical factors, confirmatory imaging diagnosis of MESCC can be hampered [9,10].

MRI is considered the most accurate imaging modality for the classification of MESCC [11,12]. MRI can provide excellent visualization of vertebral metastatic involvement and any associated malignant epidural disease and its impact on the cerebrospinal fluid space and spinal cord [13]. In cases where MRI is contraindicated, a CT myelogram is another accurate modality for assessing the degree of MESCC. Axial T2-weighted MRI images most accurately characterize the extent of MESCC via the Bilsky grading scale developed by the Spine Oncology Study Group (SOSG) [14,15]. The grading scale uses six groupings, which can be further divided into two key clinical categories; low-grade MESCC (grades 0, 1a, or 1b), which can be treated with radiotherapy (including stereotactic body radiotherapy, SBRT), and high-grade MESCC (grades 1c, or 2, or 3) which can be considered for initial surgery (e.g., separation surgery) and subsequent radiotherapy [16,17,18].

Other potential imaging modalities for MESCC include using contrast-enhanced CT without dedicated myelography or spinal protocols. Cancer patients frequently undergo CT studies of the chest and abdomen to assess the overall metastatic disease burden and response to treatment. This presents an opportunity for expedited diagnosis of MESCC and impending cord compression in asymptomatic patients or those with unclear symptoms on a background of pre-existing pain and high-dose analgesics [19]. Prior studies have assessed the utility of CT for assessing MESCC in symptomatic patients. Crocker et al. (2011) evaluated the use of staging CT in patients with suspected MESCC to identify those requiring transfer to a specialist neuroscience center for MRI [20]. In their small study of forty-four patients, CT had a sensitivity/specificity of 89%/92% for MESCC compared to the eventual MRI. Currently, the NICE guidelines on MESCC in adult patients (2008) do not recommend staging CT as a screening tool for MESCC. However, the studies analyzed did not use the latest CT scanners, and the guidelines are due to be updated soon [12,21].

This study aims to retrospectively investigate the performance of routine staging CT without myelography to detect MESCC in a group of patients who underwent MRI for suspected MESCC. CT scans performed within 30 days of the MRI will be assessed to reduce the potential for disease progression. The reference standard on CT will be provided by experienced spine radiologists using the corresponding MRI. The accuracy of CT will be determined using the existing radiology reports for the CT scans, along with a dedicated independent assessment of the scans by board-certified radiologists specializing in oncological imaging.

## 2. Materials and Methods

This study was approved by the National University Hospital, Singapore ethics review board and complied with the Health Insurance Portability and Accountability Act (HIPAA). Waiver of patient informed consent was allowed as there was minimal patient risk involved.

### 2.1. CT Scan Dataset Preparation

Retrospective retrieval and anonymization of CT studies of the thorax and upper abdomen and corresponding MRI spines from patients with known spinal column metastatic disease and suspicion of MESCC was undertaken over fourteen years from September 2007 through September 2021 at the National University Hospital, Singapore. Adults (≥18 years) were included with imaging examinations obtained across different MRI scanners (General Electric and Siemens) and CT platforms (Philips, General Electric, and Siemens platforms). Staging CT scans and corresponding MRI spine studies with a time gap of up to 1 month (30 days) were included. CT scans with intravenous contrast were used, excluding CT scans performed without contrast. In addition, staging CT scans with no corresponding MRI study (no reference standard) or CT scans performed post-MRI were excluded. MESCC on MRI was assessed using axial T2-weighted images for comparison with the staging CT. Table 1 and Table 2 provide details for the CT and MRI scanner subtypes and imaging parameters.

### 2.2. CT Scan Labeling

**Figure 1:**
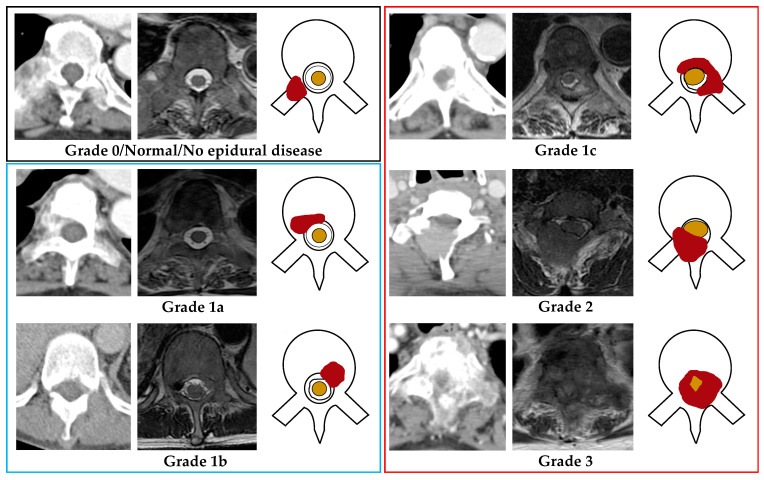
Bilsky grading for metastatic epidural spinal cord compression (MESCC) was demonstrated using axial CT, MRI (T2-weighted), and a picture for each grade (right to left). The red shaded region shows MESCC in each image. Grade 0/normal (Black outline): No metastatic epidural disease present. Low-grade MESCC (Blue outline); Grade 1a: Metastatic epidural soft tissue with no thecal sac deformity, or Grade 1b: Metastatic epidural soft tissue with thecal sac deformity but no spinal cord contact. High-grade MESCC (Red outline); Grade 1c: Metastatic epidural soft tissue touching the spinal cord with no discernible compression or displacement, or Grade 2: Metastatic epidural soft tissue cord compression with some surrounding cerebrospinal fluid still visible, or Grade 3: Metastatic epidural spinal cord compression without discernible surrounding cerebrospinal fluid. Thecal sac = black outline within the spinal canal, Spinal cord = Yellow shaded area within the spinal canal.

The staging CT dataset was manually labeled for MESCC in consensus by board-certified spine imaging specialists (AM with 7 years of experience and JTPDH with 11 years of experience) and served as the reference standard. The two radiologists labeled each axial CT image in consensus with the corresponding MRI (performed within one month) using an established visual Bilsky SOSG scale (The visual scale is provided in Figure 1).

To compare with the imaging reference standard and assess diagnostic accuracy and interobserver variability, the CT dataset was labeled independently using standardized CT windowing by three body radiologists interested in oncology imaging (YT; 3 years, PJ; 14 years, and YLT with 13 years of experience). Prior to labeling, all body radiologists were provided with a visual MESCC grading scale and reviewed practice studies providing examples of low and high-grade MESCC on MRI, and CT scans side by side. For the official focused imaging assessment of MESCC, all body radiologists were blinded to the reference standard MESCC gradings, original reports, and corresponding MRI scans.

Labeling was performed using an open-source image labeling software (LabelImg—https://github.com/tzutalin/labelImg) (accessed on 30 May 2022). This program allowed bounding boxes to be placed on each axial contrast-enhanced CT image to segment the region of evaluation around the spinal canal (C7-T1 through to the conus at T12-L3). The radiologist could click on the bounding box and change the MESCC grade. The axial CT images were evaluated using three common Hounsfield unit (HU) windows widths (W) and levels (L) for optimal radiologist assessment of MESCC; Abdomen/pelvis soft tissue windowing (W:400, L:50), bone windowing (W:1500, L:300), and additional spine soft tissue windowing (W:200, L:100). These are provided as standard settings on the GE centricity PACS (Picture archiving and communication system).

When inspecting each axial CT image, the radiologists classified MESCC using a three-grade scheme; Normal (no discernible epidural disease or Bilsky grade 0), Bilsky grades 1a or 1b amenable to first-line radiotherapy (low-grade MESCC), and grades 1c or 2 or 3, which more frequently require primary surgical decompression (high-grade MESCC) [14,16]. The visual MESCC grading scale provided to all the radiologists is shown in Figure 1. Degenerative changes (disk bulges, osteophytic change, and ligamentum flavum redundancy/ossification) producing spinal canal narrowing/stenosis were labeled and excluded from further analysis [22,23].

### 2.3. Staging CT Radiology Report Review

Along with the focused assessment for MESCC by three radiologists, we compared the accuracy of the original radiology report to the reference standard. A review of the radiology reports for each staging CT study was undertaken to assess whether there was documentation of any grade of MESCC per CT scan and at which site along the thoracic spine. Along with documentation of the MESCC grade, other terms used in the report to document MESCC included “encroachment of the spinal canal”, “Epidural disease is noted at.…”, “extension of disease into the spinal canal”, and “enhancing soft tissue noted in the spinal canal at.…”.

### 2.4. Statistical Analysis

All analyses were performed using Stata version 16 (StataCorp) with statistical significance set at 2-sided *p* < 0.05. Descriptive statistics for continuous variables were presented as mean ± SD (range) and n (%) for categorical variables. Inter-observer agreement was also calculated per axial CT image and per CT scan for both three grade (normal/no disease, low-grade, and high-grade MESCC) and two grade (normal/low-grade versus high-grade MESCC, and normal versus low/high-grade MESCC) classification using Gwet’s kappa to take into consideration a high percentage of normal/grade 0 classification [24,25]. A comparison of the radiology reports with the reference standard and radiologists using two-grade MESCC classification per CT scan (normal/low versus high-grade MESCC and normal versus low/high-grade MESCC classification) was performed. Sensitivity, specificity along with AUCs were calculated for two-grade MESCC classification.

Levels of interobserver agreement for each Gwet’s kappa value are classified as <0 = poor, 0–0.2 = slight, 0.21–0.4 = fair, 0.41–0.6 = moderate, 0.61–0.8 = substantial, and 0.81–1 = almost-perfect agreement. Furthermore, 95% confidence intervals (CIs) were presented.

## 3. Results

### 3.1. Patient Demographics, Cancer Subtypes, and MESCC Sites

An imaging data review over the fourteen-year study identified 369 patients who underwent a spine MRI for suspected thoracic MESCC. Of these, 52/369 patients (14.1%) did not have any CT study performed and were excluded from further analysis. For the remaining 317/369 patients (85.9%), there were 672 CT studies identified. Of these, 549/672 (81.7%) CT studies were excluded due to the following reasons: CT performed post-MRI (313/549, 57.0%), greater than a 30-day gap between the CT and MRI (131/549, 23.9%), CT without intravenous contrast (24/549, 4.4%), and CT images unavailable for extraction (81/549, 14.8%). Overall, there were 123 CT studies available from 101 patients for detailed analysis.

**Figure 2:**
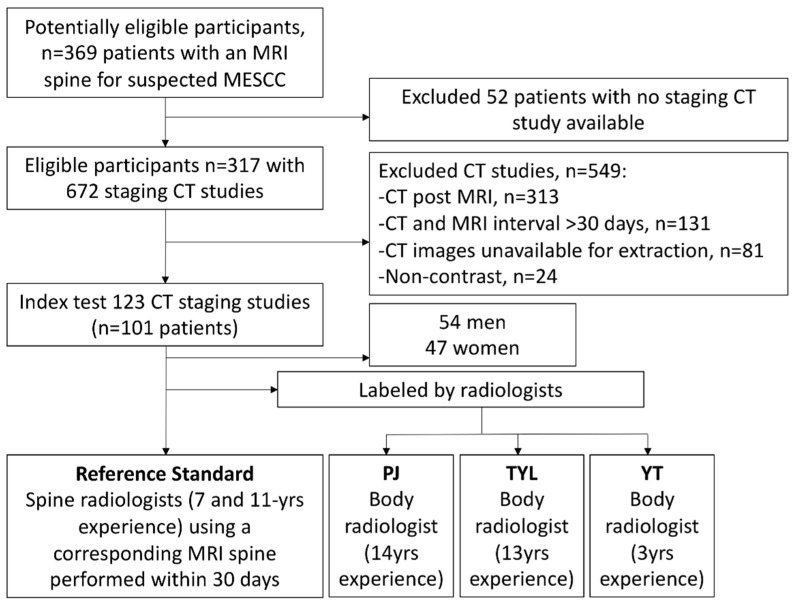
Flow chart demonstrating the overall study design with inclusions and exclusions highlighted. MESCC = Metastatic epidural spinal cord compression.

Overall, the mean age, in years, of the 101 patients was 60 ± 11.6 (SD) (range: 26–93 years). Males (54/101 patients, 53.5%) made up a greater proportion of the group than females (47/101 patients, 46.5%). Lung carcinoma (29/101 patients, 28.7%) was the most frequent malignancy, followed by breast (21/101 patients, 20.8%) and colorectal (12/101 patients, 11.9%) carcinomas. The most frequent site of MESCC along the thoracic spine was at the junction with the lumbar spine between T11-L3 (39/123 CT scans, 31.7%). Full details on the patient cancer subtypes and the MESCC locations along the spine are documented in Table 3. Figure 2 provides a study flow chart for the selection of cases.

### 3.2. Reference Standard

The number of axial CT images assessed and the corresponding MESCC grade is highlighted in Table 4. There was a high proportion of normal or grade 0 MESCC gradings for 5642/6545 images (86.2%). Low or high-grade MESCC gradings accounted for 903/6545 images (13.8%), with high-grade disease accounting for 432/903 (47.8%) images with MESCC.

On a per CT scan basis, 90/123 (73.1%) studies had at least one site of high-grade MESCC, 16/123 scans (13.0%) had at least one site of low-grade MESCC with no high-grade lesion, and 17/123 scans (13.8%) were classified as normal/grade 0 with no site of low or high-grade MESCC.

### 3.3. Three-Grade MESCC Classification

**Figure 3:**
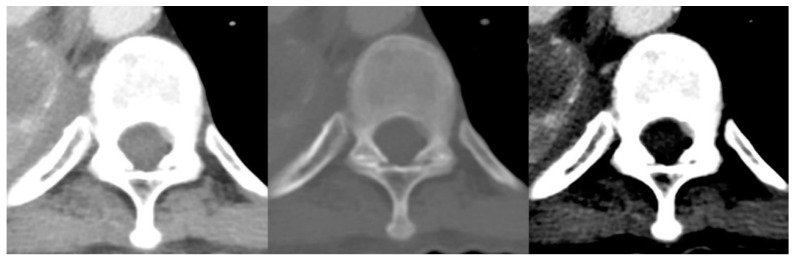
Subtle low-grade metastatic epidural disease at the left anterolateral spinal canal at T6. This was undercalled by all three radiologists and is challenging due to the paucity of bony change and location at the typical site for an epidural vein.

**Figure 4:**
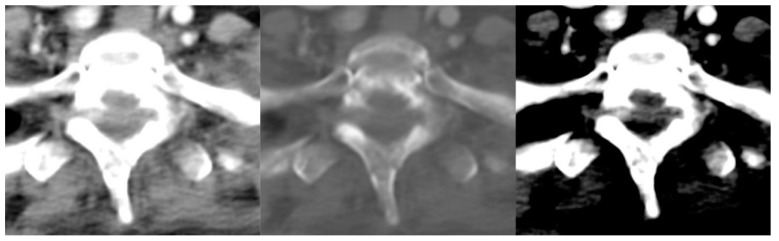
Axial CT image of the cervicothoracic junction overcalled as low-grade epidural disease by two radiologists. No epidural disease was present on the corresponding MRI (not shown). Assessment is complex due to overlap and angulation of the lower cervical and upper thoracic spine in the axial plane with background degenerative changes.

The interobserver agreements for all radiologists against the reference standard are shown in Table 5. For three-grade MESCC classification per CT image, all radiologists showed almost perfect agreement with the reference standard, with kappa values ranging from 0.927 (PJ, 95% CI 0.920–0.934) to 0.931 (YT, 95% CI 0.924–0.938). For three-grade classification per CT scan, two of the radiologists showed substantial agreement with kappa values of 0.692 (PJ, 95% CI 0.585–0.798) and 0.694 (YT, 95% CI 0.587–0.801), and TYL showed almost perfect agreement with a kappa of 0.854 (95% CI 0.777–0.931). MESCC examples on CT and MRI are shown in Figure 3 and Figure 4.

### 3.4. Two-Grade (Normal/Low Versus High-Grade) MESCC Classification

All radiologists showed almost perfect agreement for two-grade normal/low versus high-grade MESCC per CT image with kappa values ranging from 0.967 (PJ, 95% CI 0.962–0.972) to 0.978 (TYL, 95% CI 0.974–0.982) (Table 6).

Per CT image, the radiologists (PJ, TYL, and YT) showed high specificities (all >99%), whereas the sensitivities ranged from 56.48 (PJ, 95% CI 51.66–61.21) to 77.78 (TYL, 95% CI 73.56–81.61).

Per CT scan, TYL showed almost perfect agreement with a kappa of 0.905 (95% CI 0.833–0.976), while PJ and YT showed substantial agreement with kappa values of 0.653 (95% CI 0.516–0.789) and 0.688 (95% CI 0.558–0.819), respectively. The original radiology reports only had a fair interobserver agreement with a kappa of 0.213 (95% CI 0.036–0.391), which was significantly reduced compared to all radiologists (all *p* < 0.001) (Table 7).

Per the CT scan, the radiologists showed high specificities ranging from 93.94 (TYL, 95% CI 79.77–99.26) to 100 (PJ, 95% CI 89.42–100.00). Per CT scan sensitivity ranged from 74.44 (PJ, 95% CI 64.16–83.06) to 94.44 (TYL, 95% CI 87.51–98.17). Compared to the original reports, the sensitivities for all radiologists were superior, e.g., 74.44 (PJ, 95% CI 64.16–83.06) compared to only 48.89 (95% CI 38.20–59.65) for the reports (all *p* < 0.001). The AUCs for all the radiologists were also superior to the original reports, e.g., the lowest radiologist AUC of 0.872 (PJ, 95% CI 0.827–0.918), compared to 0.699 (95% CI 0.627–0.771) for the reports (all *p* < 0.001).

### 3.5. Two-Grade (Normal versus Low/High-Grade) MESCC Classification

All radiologists showed high (almost-perfect) interobserver agreement for normal/none versus low/high-grade MESCC per CT image with kappa values ranging from 0.936 (TYL, 95% CI 0.929–0.943) to 0.949 (PJ, 95% CI 0.943–0.955) (Table 8). Similar results (almost perfect agreement) were seen per CT scan with kappa values ranging from 0.866 (YT, 95% CI 0.787–0.945) to 0.947 (PJ, 95% CI 0.899–0.995) (Table 9). Per CT scan, the original radiology reports had only slight interobserver agreement with a kappa of 0.095 (95% CI −0.098–0.287, *p* = 0.333), which was significantly less compared to all radiologists (all *p* < 0.001).

Per CT image, the radiologists (PJ, TYL, and YT) showed high specificities (range 97.5–99.03), and the sensitivities ranged from 73.31 (TYL, 95% CI 70.30–76.17) to 86.82 (YT, 95% CI 84.44–88.96) (Table 8).

Per CT scan, the radiologists showed high sensitivities ranging from 91.51 (YT, 95% CI 84.49–96.04) to 98.11 (PJ and TYL, both 95% CIs 93.35–99.77). Specificities for detecting low/high-grade MESCC ranged from 64.71 (TYL, 95% CI 38.33–85.79) to 82.35 (PJ and YT, both 95% CIs 56.57–96.20). Compared to the original reports, the sensitivities for all radiologists were superior, e.g., the lowest sensitivity of 91.51 (YT, 95% CI 84.49–96.04) compared to only 44.34 (95% CI 34.69–54.31) for the reports (all *p* < 0.001). The AUCs for PJ and YT were superior to the original reports, with AUC of 0.902 (95% CI 0.808–0.997) for PJ and AUC of 0.869 (95% CI 0.772–0.966) for YT compared to 0.722 (95% CI 0.674–0.769) for the reports (*p* < 0.001 and *p* = 0.008, respectively) (Table 9).

## 4. Discussion

Early diagnosis of metastatic epidural spinal cord compression (MESCC) is important to expedite therapy and prevent paralysis. Staging CT scans are performed routinely in cancer patients and present a window of opportunity for earlier MESCC diagnosis, allowing for detailed MRI evaluation in selected patients. In this study, we assessed the performance of the original radiologist CT report for MESCC grading compared to three radiologists who performed detailed MESCC evaluations using dedicated CT windows. Two expert radiologists provided the reference standard using the corresponding MRI study performed within 30 days of the CT. For normal/none versus low/high-grade MESCC per CT scan, all radiologists demonstrated almost perfect interobserver agreement with kappa values ranging from 0.866 (95% CI 0.787–0.945) to 0.947 (95% CI 0.899–0.995), compared to only slight interobserver agreement for the original reports (kappa = 0.095, 95% CI−0.098–0.287, *p* = 0.333). Radiologists also showed high sensitivities ranging from 91.51 (95% CI 84.49–96.04) to 98.11 (95% CI 93.35–99.77), compared to only 44.34 (95% CI 34.69–54.31) for the reports.

Prior studies have shown the potential of CT to detect MESCC [20,26,27]. Pezaro et al. (2015) assessed 34 patients with known metastatic prostatic carcinoma for evidence of MESCC on staging CT [27]. They noted that MESCC was already detectable in 28/34 (80%) of the patients a median of 28 days prior to definitive spine MRI. Another study by Crocker et al. (2011) assessed the use of CT to screen for suspected MESCC to determine whether the patient required transfer to a specialist center for definitive MRI [20]. In their study, contrast-enhanced CT had a sensitivity of 89% with a high specificity of 92% for MESCC in 44 patients compared to the eventual MRI. This compares favorably to our more extensive study of 101 patients, with dedicated radiologist review showing high sensitivity of up to 98.11 and specificity of up to 82.35 for two-grade normal/none versus low/high-grade MESCC.

Accurate detection of MESCC on CT has several advantages. First, earlier detection of MESCC on routine staging CT could reduce the time to definitive MRI and treatment with improved patient outcomes, including a reduced risk of irreversible neurological injury. Suppose MESCC is detected at an earlier Bilsky grade (e.g., 1a or 1b). In that case, there is also an option for initial less invasive systemic treatment or radiotherapy, which could reduce the need for surgical intervention. Second, patients with suspected MESCC but limited access to MRI in underserved regions could undergo initial screening CT to determine the need for urgent transfer to a specialist center.

In our study, the original radiology CT reports had only slight interobserver agreement (kappa = 0.095) and reduced sensitivity (44.34) for detecting any grade of MESCC compared to a dedicated review. This poor performance is likely multifactorial. Firstly, on staging CT studies (without clinical suspicion of MESCC), radiologists will have to interrogate multiple regions, including the lungs, mediastinum, solid viscera (e.g., liver), bowel, and bony structures, including the spine. Secondly, dedicated bone windowing is typically used to assess for fractures and destructive bone metastases but does not provide adequate evaluation of the epidural space or paravertebral regions. This study’s combination of dedicated bone and soft tissue windows provided optimal assessment for any destructive bony lesion and associated epidural mass [28].

Our study has several limitations. First, we did not have multiplanar coronal and sagittal reconstructions for the CT studies and assessed the accuracy of MESCC classification using axial images alone. The use of multiplanar reconstructions, especially sagittal images similar to MRI, could improve the accuracy of MESCC assessment. Second, only CT scans with a spine MRI conducted within 1 month were analyzed to limit the potential disease progression between the studies. This limited the assessment of any time delays between the CT and MRI studies, which may have resulted in delayed MESCC diagnosis. Future studies could assess the utility of CT scans to detect epidural disease prior to the MRI using a more extended time period (e.g., up to 1 year). Expedited diagnosis of MESCC on CT could allow for earlier administration of less invasive systemic treatment or radiotherapy, reducing the need for surgical intervention [29,30]. Third, the CT scans used for analysis were identified using a surgical MESCC database. This resulted in an artificially higher proportion of positive cases with MESCC and may not represent actual day-to-day clinical practice. A potential concern is MESCC being overcalled on staging CT leading to an increase in unnecessary MRI studies and increased healthcare costs.

## 5. Conclusions

In conclusion, we assessed the utility of staging CT for the classification of MESCC. Staging CT represents a window of opportunity to triage patients for further definitive MRI. An earlier diagnosis of MESCC could allow the initiation of less invasive therapy to prevent costly surgical intervention. Radiologists using dedicated CT windows showed high interobserver agreement (kappa values ranging from 0.866 to 0.947) and sensitivities (ranging from 91.51 to 98.11) for recognition of any grade of MESCC. The original radiology reports showed relatively reduced performance (kappa = 0.095, sensitivity = 44.34), suggesting that dedicated training sessions for assessment of MESCC on CT or even the use of a deep learning algorithm for automatic classification of MESCC could improve patient care [31,32]. Future prospective studies are planned to assess the accuracy of MESCC detection on staging CT across a more extensive range of oncological patients.

## Figures and Tables

**Table 1:** CT scanner types and parameters.

Parameter	4-Slice	64-Slice	256-Slice	384-Slice	512-Slice
Pitch	1.5	1.2	0.984	0.8	0.531
Slice thickness (mm)	5	5	3	3	3
Collimation (mm)	4 × 1	32 × 0.6	128 × 0.625	192 × 0.6	256 × 0.625
kV	120	120	100	100	100–120
Reference mAs	180	200	250	200	200
Rotation time (s)	0.5	0.5	0.5	0.5	0.5

Note- kV = Kilovoltage, mAs = Milliampere-seconds. All CT scanners were located at the National University Hospital, Singapore. Scanning was conducted with patients in a supine position in a craniocaudal direction. Intravenous contrast volume for all scans = 70–100 mL depending on the size of the patient (rate of 1.2–1.5 mL/s).

**Table 2:** MRI scanner types and parameters for axial T2-weighted images.

Parameter	1.5-T	1.5-T	1.5-T	3.0-T	3.0-T
TR (msec)	3500	3500	4000	5300	5300
TE (msec)	80	80	90	100	100
Slice thickness (mm)	5	5	5	5	5
Gap (mm)	6	6	6	6	6
Field of view (mm^2^)	200 × 200	200 × 200	160 × 160	200 × 200	160 × 160
Matrix	512 × 512	512 × 512	320 × 320	512 × 512	640 × 640

Note- TR = repetition time, TE = echo time. All MRI scanners were located at the National University Hospital, Singapore. All scans were performed with patients in a supine position using a torso/body coil.

**Table 3:** Patient details and location of metastatic epidural spinal cord compression.

Characteristics	Patients (Overall *n* = 101)
Age (years) *	60 ± 11.6 (26–93)
Women	47 (46.5)
Men	54 (53.5)
Type of Cancer	
Lung	29 (28.7)
Breast	21 (20.8)
Colorectal	12 (11.9)
Renal cell carcinoma	10 (9.9)
Prostate	5 (5.0)
Hepatocellular carcinoma	5 (5.0)
Multiple Myeloma	4 (4.0)
Nasopharyngeal carcinoma	3 (3.0)
Others	12 (11.9)
Number of CT scans	123
MESCC location per CT scan	
Diffuse thoracic ^#^	32 (26.0)
C7-T2	7 (5.7)
T3-T10	28 (22.8)
T11-L3	39 (31.7)
No epidural disease	17 (13.8)

Note- MESCC = Metastatic epidural spinal cord compression. * Values presented as mean ± SD (range) for numerical and n (%) for categorical variables. ^#^ Two or more sites of MESCC.

**Table 4:** Reference standard gradings for metastatic epidural spinal cord compression.

Bilsky MESCC Grade	Per axial CT Image	Per CT Scan
Normal or 0	5642 (86.2)	17 (13.8)
Low (1a or 1b)	471 (7.2)	16 (13.0)
High (1c or 2 or 3)	432 (6.6)	90 (73.2)
Total	6545	123

Note—Values are n (%) per CT image and CT scan (~50 images). MESCC = Metastatic Epidural Spinal Cord Compression.

**Table 5:** Radiologist interobserver variability for three-grade MESCC classification on CT.

	Three-Grade MESCC (Normal, Low, and High)	
Radiologist (per image)	Kappa (95% CI)	*p*-Value
PJ	0.927 (0.920–0.934)	<0.001
TYL	0.928 (0.921–0.935)	<0.001
YT	0.931 (0.924–0.938)	<0.001
Radiologist (per scan)		
PJ	0.692 (0.585–0.798)	<0.001
TYL	0.854 (0.777–0.931)	<0.001
YT	0.694 (0.587–0.801)	<0.001

**Table 6:** Radiologist sensitivity, specificity, and AUCs for two-grade (normal/low versus high-grade) MESCC per CT image.

Radiologist	Kappa (95% CI)	*p*-Value	Sensitivity (95% CI)	Specificity (95% CI)	AUC (95% CI)
PJ	0.967 (0.962–0.972)	<0.001	56.48 (51.66–61.21)	99.90 (99.79–99.96)	0.782 (0.752–0.812)
TYL	0.978 (0.974–0.982)	<0.001	77.78 (73.56–81.61)	99.49 (99.28–99.66)	0.886 (0.863–0.910)
YT	0.973 (0.969–0.977)	<0.001	65.74 (61.05–70.21)	99.85 (99.72–99.93)	0.828 (0.800–0.856)

**Table 7:** Radiologist sensitivity, specificity, and AUCs for two-grade (normal/low versus high-grade) MESCC per CT scan.

Radiologist	Kappa (95% CI)	*p*-Value	Sensitivity (95% CI)	Specificity (95% CI)	AUC (95% CI)
PJ	0.653 (0.516–0.789)	<0.001	74.44 (64.16–83.06)	100.00 (89.42–100.00)	0.872 (0.827–0.918)
TYL	0.905 (0.833–0.976)	<0.001	94.44 (87.51–98.17)	93.94 (79.77–99.26)	0.942 (0.894–0.990)
YT	0.688 (0.558–0.819)	<0.001	77.78 (67.79–85.87)	96.97 (84.24–99.92)	0.874 (0.821–0.926)
Original reporting radiologist	0.213 (0.036–0.391)	0.019	48.89 (38.20–59.65)	90.91 (75.67–98.08)	0.699 (0.627–0.771)

**Table 8:** Radiologist sensitivity, specificity, and AUCs for two-grade (normal versus low/high-grade) MESCC per CT image.

Radiologist	Kappa (95% CI)	*p*-Value	Sensitivity (95% CI)	Specificity (95% CI)	AUC (95% CI)
PJ	0.949 (0.943–0.955)	<0.001	77.30 (74.42–79.99)	99.03 (98.73–99.26)	0.882 (0.865–0.898)
TYL	0.936 (0.929–0.943)	<0.001	73.31 (70.30–76.17)	98.49 (98.14–98.79)	0.859 (0.842–0.877)
YT	0.948 (0.941–0.954)	<0.001	86.82 (84.44–88.96)	97.50 (97.06–97.89)	0.922 (0.908–0.935)

**Table 9:** Radiologist sensitivity, specificity, and AUCs for two-grade (normal versus low/high-grade) MESCC per CT scan.

Radiologist	Kappa (95% CI)	*p*-Value	Sensitivity (95% CI)	Specificity (95% CI)	AUC (95% CI)
PJ	0.947 (0.899–0.995)	<0.001	98.11 (93.35–99.77)	82.35 (56.57–96.20)	0.902 (0.808–0.997)
TYL	0.917 (0.858–0.977)	<0.001	98.11 (93.35–99.77)	64.71 (38.33–85.79)	0.814 (0.696–0.932)
YT	0.866 (0.787–0.945)	<0.001	91.51 (84.49–96.04)	82.35 (56.57–96.20)	0.869 (0.772–0.966)
Original reporting radiologist	0.095 (−0.098–0.287)	0.333	44.34 (34.69–54.31)	100.00 (80.49–100.00)	0.722 (0.674–0.769)

## Data Availability

The data presented in this study are available on request from the corresponding author.
